# Experiences and needs of welfare benefit recipients regarding their welfare-to-work services and case workers

**DOI:** 10.1186/s12913-023-09954-y

**Published:** 2023-09-14

**Authors:** Esmee Oude Geerdink, Ranu Sewdas, Hetty van Kempen, Jaap van Weeghel, Johannes R. Anema, Maaike A. Huysmans

**Affiliations:** 1 Department of Public and Occupational Health, Amsterdam Public Health Research Institute, Amsterdam UMC, Vrije Universiteit Amsterdam, Amsterdam, 1081 BT The Netherlands; 2 Research and Statistics, Gemeente Amsterdam, Amsterdam, The Netherlands; 3 Tranzo, Tilburg School of Social and Behavioral Sciences, Tilburg University, Tilburg, The Netherlands

**Keywords:** Unemployment, Social welfare, Vocational guidance, Qualitative research

## Abstract

**Background:**

This study aimed to explore the experiences and needs of (ex-)welfare benefit recipients from a large urban municipality in the Netherlands regarding their welfare-to-work services and their case workers.

**Methods:**

Quantitative data from a client satisfaction survey that was filled out by 213 people (response rate 11%) who received welfare-to-work services was combined with results from four group interviews with a total of 15 people receiving welfare-to-work services. Verbatim transcripts from the interviews were analysed using inductive thematic analysis.

**Results:**

The survey results showed that most clients were reasonably satisfied with the welfare-to-work services they received. Four main themes emerged from the interviews: (1) experiences and needs related to the interactions between case workers and benefit recipients; (2) the need for tailored services; (3) the complicating role of the system the case workers operate within; and (4) the existence of differences between case workers regarding how strict they followed the rules and to what extent they connected with their clients on a personal level.

**Conclusions:**

Our findings show that clients were reasonably satisfied with the welfare-to-work services provided by their municipality but that there is still room for improvement. Case workers should have good social skills to build a trusting relationship with the client, welfare-to-work services should be tailored to the individual, and clear concise information should be given to welfare benefit recipients, especially with regard to what benefit recipients can expect of the municipality and the case workers, given their dual role in supporting (re-)integration to work and monitoring benefit eligibility.

**Supplementary Information:**

The online version contains supplementary material available at 10.1186/s12913-023-09954-y.

## Background

Compared to people who can earn their own income, welfare benefit recipients have a higher prevalence of physical and mental health problems, addiction, debt, and homelessness [1, 2]. Indeed, a recent systematic review in high-income countries found that health inequalities exist between welfare benefit recipients and non-recipients and that the health of benefit recipients cannot be maintained by financial aid alone [3]. Health problems can be the cause or the consequence of unemployment, and (re)employment appears to have a positive impact on health [4, 5]. Therefore, it is important that welfare benefit recipients are supported in finding a paid job.

Many OECD countries have implemented strategies to ‘activate’ welfare benefit recipients, i.e. to support and encourage individuals in their job market search, with the ultimate goal of (re)employment. Activation strategies encompass measures such as job-search assistance, training, and re-employment programmes, which people are expected to participate in in return for their benefits [6]. In the Netherlands, welfare benefit recipients who are unemployed but able to work are provided with ‘welfare-to-work’ services. A case worker employed by the municipality, that also determines eligibility for welfare benefits, oversees these services and decides which trajectory is most suitable for the client. Thereby, he or she plays an important role in the direction of the services provided and thus in the overall experience of the welfare benefit recipient.

Welfare benefits who participated in a Swedish study mentioned experiencing a loss of independence due to not being able to earn their own income [7]. Moreover, they expressed feeling ashamed about living off of welfare benefits [7]. Dutch welfare benefit recipients have also been found to experience stigma for being dependent on benefits and have indicated that the need for individualized services is not always met [8]. In addition, studies have shown that benefit recipients can feel disrespected by their welfare professionals and feel disempowered [7, 9]. These feelings of disempowerment were mainly found to occur in situations in which welfare benefit recipients had to do voluntary work as part of their activation strategy, but felt that their personal situation (personal problems, emotions) was ignored by their case worker [10]. One Australian study even found that some people felt belittled and bullied by their employment specialist, which in turn had a negative impact on their motivation and self-esteem [11].

The negative experiences found in these studies are not likely to positively contribute to the chances of finding paid employment. Yet while the above-mentioned studies described the experiences of benefit recipients, they did not investigate what benefit recipients actually need from welfare-to-work services and from their case workers. Therefore, the present study aims to explore the experiences and needs of people who receive(d) welfare benefits with respect to their welfare-to-work services and case worker in order to formulate recommendations to improve welfare-to-work services and make them more successful.

## Methods

### Setting

In the Netherlands, people who have difficulty earning their own income through employment can apply for welfare benefits at their municipality and receive a minimum income. This study took place in a large urban municipality in the Netherlands in which 39,270 citizens (6,1% of the workforce) received welfare benefits as of September 2019 [12]. In this municipality, income consultants determine whether an applicant meets the requirements to receive benefits and a case worker, who is also employed at the municipality, offers support to recipients for increasing societal participation—with the ultimate goal of finding paid employment. People who receive welfare benefits and welfare-to-work services have an obligation to do everything in their power to participate in society and, if possible, find a job [8, 13].

### Study design

This study used a mixed methods approach, combining quantitative and qualitative data to access a rich and broad understanding of the experiences and needs of people who were either eligible to receive welfare-to-work services or who had recently received these services. A client satisfaction survey was distributed by the municipality among people whose welfare benefits had ended, and quantitative data from this survey was subsequently used to investigate recipients’ satisfaction with welfare-to-work services and with their case workers. Qualitative data was gathered during group interviews with current welfare-to-work recipients to achieve a more in-depth understanding of the survey results and to study benefit recipients’ needs concerning the services and case workers.

The Medical Ethical Committee of the Amsterdam University Medical Centres approved this study and concluded that it is not subject to the WMO (Medical Research Involving Human Subjects Act) (2019.460). Written informed consent was obtained from all participants of the group interviews.

### Client satisfaction survey

#### Data collection

In this municipality, an online survey is sent out four times per year to determine satisfaction with services among former welfare-to-work recipients who have found a job. The survey is sent out to citizens whose welfare benefits have ended in the past three months (i.e., since the last survey was sent out), usually because they have found a paid job. For this study we used the results of two surveys that were sent to 1889 clients and filled in by 213 (response rate 11%) between July and December 2019.

#### Me﻿asurem﻿ents

Certain background characteristics were available for the people who filled in the survey, including age brackets, the number of years they had received welfare benefits, and their current work status. For this study, 32 statements from the survey were used (leaving out 4 statements that were not relevant for our research question). The statements could be answered on a 1–5 scale, ranging from ‘totally agree’ to ‘totally disagree’. In addition, one statement was used in which satisfaction with the welfare-to-work services had to be rated on a scale from 1 to 10 (see Appendix 1). The statements included general topics related to the support benefit recipients received (4), topics regarding their relationship with the case worker and other professionals (12), the clarity of information provided by the case worker and the municipality (11), and the support they received during job applications (5).

#### Data analysis

Chi squared tests were performed to assess differences between respondents and non-respondents of the survey and to determine whether there was a response bias. For each statement in the survey, we determined the number and percentage of people who agreed. We then created a categorical variable to determine agreement: disagree (answering options “disagree” and “totally disagree”), neutral (“neither agree or disagree”), and agree (answering options “agree” and “totally agree”). For the question regarding general satisfaction, the mean and standard deviation were calculated.

### Group interviews

#### Recruitment and participants

Participants were eligible for participation in the group interviews if they were 27 years of age or older, were receiving welfare-to-work services at the moment or had in the past (which had ended max. 6 months ago), and were able to work (even with poor job prospects, or when they were not able to work directly but training beforehand was required). The age limit of 27 years or older was chosen because in this municipality, clients below 27 years old are classified as ‘young adults’ and receive coaching from different teams than adults, which is most often focused on starting education instead of work. Likewise, we only included people who were able to work because people who are considered not able to work receive a different kind of support, not focused on participation in paid work.

Participants were chosen using convenience sampling and approached by a researcher during group trainings provided by the municipality as part of the welfare-to-work services. One of the researchers (RS) visited these trainings to give oral information about the research and invite participants for the group interviews. Since we know from experience that this often does not result in a large response, we used an additional recruitment strategy. Therefore, we also asked case workers to ask their clients if they are willing to participate. Case workers were approached by the researchers via email and were asked to send an email to participants and ask them whether they were interested in participating in the interviews. Case workers were made aware that it was important to not only recruit clients who had a ‘successful’ trajectory. Because a power disadvantage exists between the case worker and client, case workers were also instructed to carefully explain to clients that participation was voluntary and that whether they participated or not would not affect their trajectory at the municipality. Moreover, who participated or not and what was said by whom during the interviews was kept confidential. This was again explained by the researchers when clients said they wanted to participate. Since many case workers were approached and not all of them responded, we were unable to determine how many participants were invited and refused to participate or their reasons for not participating.

When clients indicated they wanted to participate in the study, they received an information letter that explained the aim of the study and practical information regarding the group interviews, e.g. time investment and location. The information also explained possible advantages (i.e. giving your opinion on the welfare-to-work services) and disadvantages (time investment, traveling to the location). Finally, the letter explained that participation in the study was voluntary and participants could stop participation at all times. Moreover, it contained an explanation on storage of the data and privacy. In the letter it was also made clear that anything participants said during the group interviews would be handled confidentially and results would only be shared in an anonymous way. All participants received a €25 gift card as a gratitude token.

#### Data collection

In September 2019, four semi-structured group interviews took place varying in length from 1.5 to 2 h. All interviews were conducted by a researcher (RS) and a co-interviewer who was an experience expert (i.e., who had received welfare benefits and welfare-to-work services in the past). The co-interviewer was involved in the design of the topic list, took notes and was an active participant in the interviews, e.g. by asking (clarifying) questions. We added the presence of an experience expert because we know from experience that this has a positive effect on compiling an appropriate topic list with language that suits the participants and on creating a save environment for participants of the group interviews.

Three interviews took place in a municipality office and one took place at a training location during a training that was attended by people who receive welfare-to-work services. At the interview during the training, two independently contracted trainers were present and took part in the conversations, but their input was excluded from the analyses. We believe that the presence of these external trainers did not change the topics that were discussed, because the interviewers used a topic list and made sure the input of the external trainers was limited. We also believe their presence did not put pressure on participants to give different and perhaps more positive answers. The interviewers made sure the interviews were conducted in a safe setting, it was clear for all participants that everything that was discussed was confidential and it was clear for participants that the trainers were from an external agency and were therefore not affected by their responses, or in close contact with the case workers.

A topic list was used to guide interviews that included questions regarding experiences (e.g., “can you describe your experiences with the provided services and your case worker” and “which parts of the services did you perceive as pleasant and which parts did you perceive as unpleasant”) and needs (e.g. “what characterizes good or bad support” and “what should a case worker do and what shouldn’t a case worker do”) (see Appendix 2). No additional themes came up in the last two interviews, which we see as an indication of data saturation. All interviews were audio recorded and transcribed verbatim.

Before starting the interviews, all participants signed an informed consent form that states participation is voluntary and they could withdraw consent at any time.

#### Data analysis

We analysed the data using inductive thematic analysis, in which we aimed to provide a rich description of the data [14]. We did not start data analysis until all group interviews were completed. First, to familiarize ourselves with the data, two researchers (RS, EO) read all transcripts and a summary was made by one of them (RS) based on first impressions. One of the group interviews was then coded using open coding by those two researchers separately, and when the codes were compared, we found no important differences. We then started developing initial themes, and after discussing the codes and initial themes among the research team, all other transcripts were coded by one of the researchers (EO). During coding and developing initial and final themes, EO had regular discussions with RS and MH, and all codes and themes were also discussed regularly within the entire research team.

## Results

### Experiences – results from the client satisfaction survey

Between July and December 2019, a client satisfaction survey was filled out by 213 clients; 56% of the respondents were between 27 and 50 years old, and the majority had received welfare benefits for more than a year (56%). More than 90% of the participants were employed in a regular paid job at the time of completing the survey. Table 1 shows the descriptive statistics of participants who filled in the survey. The characteristics of non-respondents were comparable to respondents, except for age. Chi squared tests showed that the distribution across age brackets was significantly different between respondents and the total group of people the survey was sent to: those who responded to the survey were slightly older than the total group of people.

**Table 1:** Descriptive characteristics of survey respondents and non-respondents

	Participants (n = 205)^1^	All citizens the survey was sent to (n = 1889)
Age (n, %)^2,3^		
< 27 years 27–50 years > 50 years	20 (10%) 114 (56%) 71 (35%)	315 (17%) 1228 (65%) 346 (18%)
Years of receiving welfare benefits		
(n, %) < 0,5 year 0,5–1 year 1–2 years > 2 years	51 (25%) 38 (19%) 29 (14%) 87 (42%)	570 (30%) 316 (17%) 306 (16%) 697 (37%)
Employment status (n, %)		
Regular paid job Subsidized job Self-employed	189 (92%) 4 (2%) 12 (6%)	1717 (91%) 29 (2%) 143 (8%)

^1^Descriptive data missing for eight people

^2^These age brackets correspond to the age categories the municipality uses to tailor their services

^3^ The age spread was significantly different between participants and the overall group of citizens the survey was sent to: X^2^ = 32.810, p = 0.000

Table 2 shows the results of the most important items of the client satisfaction survey. Results of additional items can be found in appendix 3. Participants rated their general satisfaction with the welfare-to-work services at an average of 6,6 (SD = 2,7) on a scale from 1 to 10. About half of the participants (51%) agreed that the meetings with their case workers were necessary for finding a job and that the support of the municipality helped them to find a job.

Most participants agreed that their case worker was kind (76%) and that they were treated kindly (69%) and with respect (67%) during their welfare-to-work trajectory. However, less than 60% of the participants agreed that their case worker (59%) and other professionals (47%) knew enough about their personal situation and took all aspects of their personal situation into consideration, and only 47% of participants agreed that their case worker tried to find a job that suited them.

Out of the 213 participants, 51 people (24%) received support preparing for one or more job interviews. Of these people, the majority was satisfied with this support; 82% agreed that they were able to prepare well for the interview(s) with their contact person and that they learned a lot from that.

**Table 2:** Results of the client satisfaction survey (n = 213)

*General statements*	Mean (SD)		
Can you grade on a scale from 1 to 10 your general satisfaction with the welfare-to-work services you received from the municipality?	6,6 (2,7)		
	**Disagree** (n (%))	**Neutral** (n (%))	**Agree** (n (%))
My case worker was kind.	12 (6%)	39 (18%)	162 (76%)
My case worker tried to find work that suits me.	56 (26%)	56 (26%)	101 (47%)
I felt treated kindly during the welfare-to-work trajectory.	23 (11%)	42 (20%)	148 (69%)
Other professionals (e.g., people who provided trainings) knew enough about my personal situation.	49 (23%)	64 (30%)	100 (47%)
The purpose of the meetings was always clear to me.	32 (15%)	38 (18%)	143 (67%)
It was clear to me which (financial) services I can (still) use now that I have a job (such as reimbursement of travel expenses, day care).	68 (32%)	48 (23%)	97 (46%)
***Statements regarding the support for job interviews or applications***			
Did you receive help from (someone who works at) the municipality for the preparation for your job interview(s)?	Yes: 51 (24%) No: 162 (76%)		
I was able to prepare well for the job interview(s) with my contact person.	1 (2%)	8 (16%)	42 (82%)

### Experiences and needs – results from the group interviews

In total, 14 welfare-to-work services recipients agreed to participate in the interviews. Four participants were recruited by their case worker and ten by the researcher. Four group interviews with 2–6 participants were planned in consultation with the participants. Table 3 shows the self-reported characteristics of the participants of the group interviews. Most participants were female (86%) and almost half of the participants were unemployed (43%) at the time of the interview. The length of receiving welfare-to-work services among participants varied from a couple of weeks to eight years. Participants had taken part in various programs as part of their welfare-to-work services, such as job application trainings or internships. The majority of participants had taken part in more than one of these programs. A description of the characteristics of participants per group interview can be found in appendix 4.

**Table 3:** Self-reported characteristics of the participants of the group interviews

	Participants (n = 14)
Age in years (median, range)	40,5 (28–58)
Female (n, %)	12 (86)
Work status (n, %)	
- Unemployed	6 (43)
- Volunteer work, work programme	4 (29)
- (Self-) Employed, in education	4 (29)
Educational level (n, %) *	
- Low	4 (29)
- Medium	4 (29)
- High	6 (43)
Time of receiving welfare-to-work services	< 4 weeks to > 8 years

* Low = primary education, first three years of senior general secondary education or pre-university education, prevocational education, and lower secondary vocational training

Medium = upper secondary education, basic vocational training, vocational training and middle management and specialist education

High = associate degree programs, higher education bachelor’s programs, master’s degree programs and doctoral degree programs

#### General experiences regarding the participants’ situations

Almost all participants mentioned that they experience physical or psychological health problems or have problems in other areas of life (for example regarding debts or housing). Most participants mentioned wanting to ‘do something’, and not wanting to ‘sit at home’. Some participants mentioned that it made sense to them to have to do something in return for receiving welfare benefits and that they feel that this is fair. Some participants also mentioned that they appreciated it when they were given some responsibility in their trajectory to work because this made them feel taken seriously, which in turn gave them motivation and self-confidence.

#### Interaction between recipients and case workers

According to participants, a case worker should have good social skills: they should be empathetic, listen sincerely, and be able to give clear and concise information. It was greatly appreciated by participants when they felt their case worker was sincerely interested and really listened to them: what are their talents, abilities, ambitions, interests, strengths, and weaknesses? What kind of work would they like to do? On the other hand, participants could become frustrated or angry when they felt that their case worker was not empathetic or trying to understand the client’s situation.

“Yes, and that is why I got so angry, due to the lack of empathy. Because this guy was like ‘you should keep your mouth shut, this is what happened’. And I said: ‘There was no clear decision, I was not informed at all and suddenly a part of my benefits was taken away’. Well, when that happens and the case worker can’t even say ‘Okay, I understand, it was very unfortunate’, yes of course I get angry then!” (FG4R1).

According to participants, it is really important that a case worker is able to create a trusting and equal relationship, because the obligation to share personal information can make people feel very vulnerable and uncomfortable, and people need to feel safe in order to be able to talk about personal issues such as debts or health problems.

“Well, you go there (for the first time) and you think: shit, I have to start working. Oh no, I have to do ‘gardening services’. Those are fears you have when you go there, because you also know that you don’t get money for no reason. That makes sense, and it is nice that you can get support. But that doesn’t mean that you should expose yourself completely, but you do feel like that, which makes the situation a bit frightening in the beginning. So, I think that the case worker can get a lot more done when they have a social and orienting conversation the first time”. (FG1R1)

Many participants expressed that they want their case worker to approach them in a positive way, without any prejudices or judgement. Some participants mentioned that they felt their case worker did not take them seriously or respect them or that their case worker seemed to have the impression that welfare benefit recipients do not want to work in general. It seems that most participants felt that there was some stigma regarding being a welfare benefit recipient, which made them feel less confident and motivated. Participants also wanted their case workers to give them a compliment every now and then, in order to increase their confidence and motivation.

“He took me seriously, he saw me, yes, I feel like this sounds very emotional, but he saw me as a person and not as a client or fool. He was also a bit amicable, just fun, when I went somewhere he would call me and ask how it went. I can also just call him on his cell phone […]. He was just a nice guy. Which also makes you try harder, I think, when somebody acts like that you get a lot more done than when somebody acts superior and treats people (as) pathetic. That is really not good”. (FG1R1)

#### The need for tailored services

Participants mentioned the importance of case workers considering their personal situations during the welfare-to-work trajectory, including physical or psychological health, financial, or social problems. Participants also said they appreciated it when a case worker was able to ‘think outside of the box’ and perform actions that were not necessarily part of doing their job.

“Also, I missed an appointment and at the next appointment she said ‘Okay, I won’t be too strict regarding this, because I know why it happened and what the problem is’. And then she advised me to go somewhere where they can help you with financial problems. But I didn’t do that. She then thought about what the easiest solution would be for me […]. Those small things: the regular elements of the welfare-to-work services she could use did not work, but she thought along with me”. (FG3R2)

Most participants appreciated it when case workers gave advice, for example regarding financial problems or taking part in trainings. However, participants made it clear that the case worker should be careful when giving advice because this can also be perceived by clients as telling them what to do. However, the interviews did not make it clear exactly how a case worker can ensure that their advice is perceived as positive.

#### Role of the system

Some participants mentioned that the system the case workers operate within might force case workers to push their clients, and they experienced a power dynamic between themselves and the case worker. Participants thought that case workers are pressured by management to push clients to apply for a job, and mentioned thinking that case workers need to reach targets (i.e., they have an obligation to make sure a certain number of people start work within a certain period of time). Since participants are dependent on their welfare benefits and these can be stopped by the municipality if they do not meet their obligations, participants felt dependent on their case worker, which could increase the feeling of a power dynamic.

“[…] the government wants everybody to have a job, if you do not have a job you have to go and clean the streets. They say: ‘you have to, and you cannot refuse’. Because if you refuse, you will also get punished”. (FG1R3)

In two of the group interviews, participants mentioned that the focus in the system and the focus of the trajectories should not only be on finding a paid job but on societal participation as well. Some participants mentioned they were not allowed to do voluntary work or felt they were not being appreciated for it, even though they felt this was a great contribution to society. Some participants experienced barriers to starting a regular paid job that they said did not stop them from doing voluntary work, e.g. because you cannot go to a paid job only incidentally.

Many participants mentioned that when there was an obligation to apply for a certain number of jobs each week this was not productive, because this caused them to apply for jobs which weren’t suitable. This in turn led to many rejections, which participants said could cause a decrease in confidence and motivation.

#### Differences between case workers

From the quotations and description above, we can conclude that participants experienced differences between case workers and the ways they interacted with the welfare-to-work recipients. Participants mentioned thinking that not everybody was treated in the same way and that treatment may be dependent on the particular case worker they have. For example, there seemed to be differences regarding how hard case workers pushed welfare benefits recipients and to what extent they connected on a personal level with the recipient and took their interests, personal lives, and social or health problems into account. Moreover, participants mentioned that the welfare benefit recipients themselves could also influence the way they were treated by the case worker—somebody who was less assertive might be more easily pushed to participate in trainings or apply for jobs that might not suit them than somebody who was more confident and assertive.

#### Recommendations

Table 4 shows an overview of the clients’ recommendations for case workers and the organization of welfare-to-work services, that we formulated based on the experiences and needs of welfare service recipients that were most commonly mentioned in the focus groups. It must be noted that these recommendations are not necessarily feasible, because municipalities are limited by financial aspects and legal frameworks; the recommendations are more of a description of the ideal situation based on the experiences and needs of clients that were collected during these group interviews.

**Table 4:** Recommendations based on the experiences and needs expressed by welfare service recipients in group interviews regarding welfare-to-work services and their case workers

***Recommendations for skills and behaviour of case workers*** *According to clients, a case worker should:*	
***1***	*have good social and communication skills, to;* - *get to know the client on a personal level* - *create a trusting, safe and equal relationship*
***2***	*tailor the welfare-to-work service they provide to the individual client;* - *clients’ personal situation and interests should be taken into account when looking for a job*
***3***	*work systematically and methodologically;* - *each case worker should operate in the same way* - *case workers should treat all their clients in the same way*
***Recommendations for the system the case worker operates in*** *According to clients, the system should*:	
***4***	*be more supportive, and less demanding;* - *clients should not have the obligation to apply for a certain number of job* - *clients should be allowed to perform voluntary work instead of a regular paid job, when the client wishes to do so*

## Discussion

This study explored the experiences and needs of people who receive(d) welfare benefits in a large municipality in the Netherlands with regard to the welfare-to-work services they receive(d) and their case worker. Quantitative data showed that most participants who found a job were reasonably satisfied with the welfare-to-work services they received but that there is room for improvement. The qualitative data gave further insight into the experiences and needs of people receiving welfare benefits, which could be used to improve the welfare-to-work services: first, regarding the interaction between benefit recipient and case workers positive experiences (such as case workers who really listened to their clients) but also negative experiences (such as a lack of empathy from the case worker) were found. Second, clients mentioned the need for welfare-to-work services to be tailored to the individual (i.e., the case worker should take personal circumstances, interests, motivation, talents, and abilities into account). Third, it became apparent that the system the case workers operate within seems to complicate their ability to meet the needs of clients. Finally, it was discussed that unwanted differences between case workers regarding the way they enforce the law and interact with their clients exist. Based on these findings, we formulated recommendations that reflect the needs of clients and could thereby contribute to improving the welfare-to-work services, which in turn can lead to an increase in work participation amongst people who receive these services. These recommendations are related the themes we found in the group interviews; (1) case workers should have good social and communication skills, (2) welfare-to-work services should be tailored to the individual, (3) the system should be more supportive and less demanding, and (4) case workers should work systematically and treat all clients in the same way.

In the interviews, positive as well as negative experiences regarding the interaction between recipients and case workers were described. These experiences corresponded with the quantitative results, which showed that the majority (76%) of the participants agreed that their case worker was kind and felt that they were treated kindly during the entire welfare-to-work trajectory (69%), but the need for tailored services was not always being met. For example, only 47% of clients agreed that their case worker tried to find a job that suited them, which was also often mentioned as a negative experience in the interviews. The remaining themes from the interviews, i.e., the impact of the system the case worker operates in and the differences between case workers, were not addressed in the satisfaction survey. It is interesting to note that the survey showed that it is not always clear for clients what to expect from the municipality and what their rights and obligations are during the welfare-to-work trajectory or when starting a job, yet this was rarely mentioned in the interviews. Although it was not explicitly mentioned in the interviews, a lack of clarity regarding the system and the role that case workers and benefit recipients themselves play in the system, might have led to negative experiences. For example, the negative experience of being pushed by a case worker can be due to lack of clarity regarding the system, or having expectations about voluntary work that are not realistic might lead to a negative experience. Whether an increase in (clarity of) information can improve this should be further explored in future research.

As in our study, previous Dutch studies that examined the experiences of welfare-to-work clients who were asked to do voluntary work by their activation workers (‘workfare volunteering’) also found that clients expressed the need for appreciation and mentioned the importance of taking the clients’ skills, interests, and experiences into account when looking for a (voluntary) job [9, 10, 15]. In contrast to our study, however, most of those clients indicated that they wanted to find a paid job after volunteering for a limited period of time, whereas in our study some participants mentioned that they preferred to participate in voluntary work instead of a paid job. The most important reason that some participants in the present study did not want paid employment seemed to be experience of some form of pressure in a regular paid job (for example regarding absence) that they do not experience in voluntary work. While the population in these studies were comparable to ours, the difference in results could perhaps be explained by the fact that this wish for voluntary work was mainly expressed by women with the care for young children, of whom multiple were present in one of the group interviews in our study.

We also compared the results of or study with previous literature on Supported Employment (SE) specialists, since some tasks of a case worker are comparable to those of Supported Employment (SE) specialists, who indeed also support their clients in finding a job. We found that our results regarding the importance of a good and trusting relationship and factors such as empathy, respect, listening, and being non-judgmental being important in this relationship were in line with results from qualitative studies among SE specialists and their clients [16–20]. In contrast to our findings, however, these studies did not describe experiences of clients who felt being pushed towards a specific job or experiences of a hierarchic relationship. These differences may be explained by the differences in the role of the case workers in the present study compared to that of SE specialists; case workers are employed by the organization that also decides whether clients are eligible to receive welfare benefits, whereas SE specialists work independently from organizations in charge of the provision of benefits and therefore the preferences of clients can always be the first priority. In that regard, the role of the case worker in the municipality may be more comparable to that of the healthcare worker in the worker’s compensation system (i.e., the occupational or insurance physician) who are not only responsible for healthcare but also play a role in the justification for receiving sickness benefits. Indeed, similar to the participants in our study, literature shows that injured workers sometimes feel pressured by their health care providers, and health care providers who support and respect the injured worker and their individual needs are positively appreciated [21, 22]. From these comparisons, it can be concluded that the context and the system in which the professional who oversees the work trajectory operates in has an important impact on the relationship between the professional and the client and therefore on the success of the trajectory.

### Strengths and limitations

This study had several strengths and limitations. The first strength is that we used a combination of qualitative and quantitative data to obtain a broad overview and deeper understanding of the experiences and needs of people who receive welfare-to-work services. Second, we not only explored the experiences of welfare benefit recipients but also addressed what they feel they need from the services and their case workers to improve these experiences.

A limitation of the present study is that selection bias has likely occurred in the survey, since it was sent only to participants who had found a job. It is likely that those who did not find a job had different (and perhaps less positive) experiences. In addition the response rate of the survey was low, however, it is not clear how this may have affected the results. In general, older individuals responded to the survey, which may have led to more positive experiences since older adults in general have more difficulty finding employment. At the same time, it could be that clients with more negative experiences had more of a need to share them by responding to the survey. To compensate, we made sure that in the interviews both perspectives (i.e., of those who had already started a (voluntary) job or training and those who had not) were represented, and we included both older and younger participants. Still, some of the participants in the interviews were invited by their case worker, and it is likely that participants with more positive experiences and a good relationship with their case worker were invited and/or agreed to participate. In general, we have found that this population is extremely hard to reach for participation in research. We do, however, believe that the results of the survey in combination with the group interviews are a good step in exploring the satisfaction of different elements of welfare-to-work services and in identifying elements that need improvement. A final limitation of this study is the limited number of participants in the group interviews: as mentioned before, it also was very difficult to recruit participants for the group interviews and therefore the original plan of six participants per focus group was not carried out. However, in analysing the data no new themes came up in the last two interviews and, therefore, we still feel that data saturation was reached.

### Implications for practice and research

Several implications for practice can be deduced from the results of this study. First, participants expressed the need for a case worker who has good social and communication skills. Even though many case workers already have these competences, selecting and coaching or training case workers on these skills to improve their relationships could be beneficial. Participants also expressed needs which are linked to the behaviour of the case workers (e.g., a case worker should get to know the client, give compliments, be sensitive to clients’ cultural backgrounds, and take the personal situation and interests of the client into account). It could be argued that case workers should be coached and trained in this behaviour, but the context in which the case workers operate does not always allow the case workers to meet all of the needs that were expressed in the interviews. For example, considering the interests of the client may not be possible when the client specifically wants to do voluntary work instead of a paid job but the system requires that case workers support and coach welfare benefit recipients to find a paid job whenever possible. Therefore, it seems of utmost importance that clients are provided with clear information about their rights and duties and the role, possibilities, and limitations of the case worker and the municipality. It is likely that clarity about what to expect might increase their satisfaction with the welfare-to-work services and case workers, regardless of the rules that are set by the system.

Further research should examine whether the provision of clear information about rights and duties and the role of the case worker can indeed contribute to more realistic expectations among clients and increase their satisfaction. In addition, future research should also examine whether the relationship between case workers and welfare service recipients can be improved by changing the behaviour of the case worker and thereby the interaction between the case worker and welfare service recipient; whether changes in the welfare-to-work system are necessary; or whether both aspects should be addressed since they are intertwined. Additionally, it is recommended that further research on the satisfaction with welfare-to-work services amongst a broader group of participants (i.e., clients who still receive welfare benefits and have not started a (paid) job and clients in different municipalities) is performed. A broader group of stakeholders, such as case workers and their managers, as well as participants in different phases of their welfare-to-work trajectory, should be interviewed to further discuss possible solutions for the aforementioned dilemmas.

## Conclusions

In general, welfare benefit recipients in this municipality were reasonably satisfied with their welfare-to-work services. Participants who received welfare-to-work services in the municipal setting described both positive and negative experiences, which showed there is room for improvement. Further research is needed to examine how to improve the provision of information to welfare benefit recipients and the relationship between welfare benefit recipients and their case workers, to eventually better meet the needs of welfare benefit recipients and thereby improve the effectiveness of welfare-to-work services.

Statements & Declarations.

### Electronic supplementary material

Below is the link to the electronic supplementary material.

Supplementary Material 1

Supplementary Material 2

Supplementary Material 3

Supplementary Material 4

## Data Availability

The data that support the findings of this study are available on reasonable request from the corresponding author EO. The data are not publicly available due to them containing information that could compromise research participant privacy.

## Declarations

### Authors’ information

Authors RS and HvK are employed at the city of Amsterdam. Author EO is an external employee at the city of Amsterdam. Author JRA holds a chair in Insurance Medicine on behalf of the Dutch Social Security Agency and is shareholder of the VUmc Spin-off company Evalua Nederland B.V. and IkHerstel LtD. Author JvW was scientific director of Phrenos during the conduct of the study.
