# Supplementary material for: Experiences and needs of welfare benefit recipients regarding their welfare-to-work services and case workers
Source: BMC Health Serv Res. 2023 Sep 14;23:990. doi: 10.1186/s12913-023-09954-y (PMC10502984; doi:10.1186/s12913-023-09954-y)
Supplement: Supplementary file 1 — Supplementary Material 1 [file 12913_2023_9954_MOESM1_ESM.docx]

**Appendix 1 – Client satisfaction survey**

- Can you grade your general satisfaction with the welfare-to-work services you received from the municipality on a scale from 1 to 10?

General statements

- I needed the meetings with my case worker in support of finding a job
- Thanks to the support I received of the municipality I have found a job
- The municipality has stimulated me enough to find a job
- I have great confidence in my future now that I have a job (again)

Statements regarding the relationship with the case worker

- I always felt comfortable during the meetings with my case worker
- My case worker was kind
- My case worker motivated me to find a job
- My case worker gave me confidence to return to work
- My case worker thought along with me about the possibilities for work
- My case worker took all aspects of my personal situation into consideration
- My case worker tried to find work that fit me

Statements regarding the relationship with other professionals

- I felt treated with respect during the welfare-to-work trajectory
- I felt treated kindly during the welfare-to-work trajectory
- Other professionals (e.g., people who provided trainings) knew enough about my personal situation
- My contact people were always easy to reach in cases of questions
- My contact people always kept their appointments

Statements regarding the clarity of information provided by the case worker and the municipality

- The purpose of the meetings was always clear to me
- It was always clear to me how to prepare for the meetings
- The meetings provided me clarity on what was expected of me during the trajectory of finding a job
- The meetings provided me with clear information on what I could expect from the municipality regarding support in finding a job
- My case worker informed me well about my rights and duties when finding a job
- It was clear to me that I had to inform my case worker about my new job
- The consequences of my new job for my welfare benefits were clear to me
- The consequences of my new job for the financial arrangements which I (am allowed to) receive were clear to me
- It was clear to me which services I could (still) use now that I have a job (such as reimbursement of travel expenses, day care)
- It was clear to me that I could always call my case worker in case of issues during the first months of my job
- My case worker informed me well about the consequences of finding a job on my welfare benefits

Statements regarding the support for job interviews

- Did you receive help from (someone who works at) the municipality for the preparation for your job interview(s)?
- I was able to prepare well for the job interview(s) with my contact person
- My contact person was kind enough to wish me luck with my job application(s)
- My contact person showed interest in how my job interview(s) went
- I learned a lot from the preparations for my job interview(s)
