# Supplementary material for: Experiences and needs of welfare benefit recipients regarding their welfare-to-work services and case workers
Source: BMC Health Serv Res. 2023 Sep 14;23:990. doi: 10.1186/s12913-023-09954-y (PMC10502984; doi:10.1186/s12913-023-09954-y)
Supplement: Supplementary file 2 — Supplementary Material 2 [file 12913_2023_9954_MOESM2_ESM.docx]

**Appendix 2 – Topic list group interviews**

**Introduction round**

- Goal of the interview: to share your own experiences and/or opinions regarding the welfare-to-work services and regarding the case worker. We want to know how you experienced this: what went well, what can be improved?
- Name, age, occupation, duration of receiving welfare benefits

**Questions**

- What do welfare-to-work services and interactions with your case manager look like at this moment?
- Which aspects of the services are pleasant and which are less pleasant?
- What are the features of good welfare-to-work services and of a good case worker, and what should not be done?
- What does an ideal conversation with a case manager look like?
- Which (personal) aspects are of importance for the services and in the conversations, and which are not?

**Rounding up**

- Do you have concrete tips or propositions to improve the welfare-to-work services?
- Are there topics that we did not discuss, but you would like to add? This can be anything that comes to mind!
