# Supplementary material for: Experiences and needs of welfare benefit recipients regarding their welfare-to-work services and case workers
Source: BMC Health Serv Res. 2023 Sep 14;23:990. doi: 10.1186/s12913-023-09954-y (PMC10502984; doi:10.1186/s12913-023-09954-y)
Supplement: Supplementary file 3 — Supplementary Material 3 [file 12913_2023_9954_MOESM3_ESM.docx]

**Appendix 3 – Additional of the client satisfaction survey**

| ***General statements (n=213)*** | **Disagree** (n (%)) | **Neutral** (n (%)) | **Agree** (n (%)) |
| --- | --- | --- | --- |
| I needed the meetings with my case worker in support of finding a job. | 59 (28%) | 45 (21%) | 109 (51%) |
| Thanks to the support I received of the municipality I have found a job. | 66 (31%) | 38 (18%) | 109 (51%) |
| The municipality has stimulated me enough to find a job. | 35 (16%) | 55 (26%) | 123 (58%) |
| I have great confidence in my future since now that I have a job (again). | 20 (9%) | 50 (23%) | 143 (67%) |
| ***Statements regarding the relationship with the case worker*** | |  |  |
| I always felt comfortable during the meetings with my case worker. | 28 (13%) | 40 (19%) | 145 (68%) |
| My case worker motivated me to find a job. | 30 (14%) | 53 (25%) | 130 (61%) |
| My case worker gave me confidence to return to work. | 27 (13%) | 53 (25%) | 133 (62%) |
| My case worker thought along with me about the possibilities for work. | 30 (14%) | 53 (25%) | 130 (61%) |
| My case worker took all aspects of my personal situation into consideration. | 42 (20%) | 46 (22%) | 125 (59%) |
| ***Statements regarding the relationship with other professionals*** | |  |  |
| I felt treated with respect during the welfare-to-work trajectory. | 26 (12%) | 45 (21%) | 142 (67%) |
| My contact people were always easy to reach in cases of questions. | 32 (15%) | 44 (21%) | 137 (64%) |
| My contact people always kept their appointments. | 24 (11%) | 42 (20%) | 147 (69%) |
| ***Statements regarding the clarity of information provided by the case worker and municipality*** | |  |  |
| It was always clear to me how to prepare for the meetings. | 31 (15%) | 41 (19%) | 141 (66%) |
| The meetings provided me clarity on what was expected of me during the trajectory of finding a job. | 38 (18%) | 38 (18%) | 137 (64%) |
| The meetings provided me with clear information on what I could expect from the municipality regarding support in finding a job. | 48 (23%) | 43 (20%) | 122 (57%) |
| My case worker informed me well about my rights and duties when finding a job. | 41 (19%) | 60 (28%) | 112 (52%) |
| It was clear to me that I had to inform my case worker about my new job. | 19 (9%) | 25 (12%) | 169 (79%) |
| The consequences of my new job on my welfare benefits were clear to me. | 25 (12%) | 18 (8%) | 170 (80%) |
| The consequences of my new job for the financial arrangements which I (am allowed to) receive were clear to me. | 50 (23%) | 39 (18%) | 124 (58%) |
| It was clear to me that I could always call my case worker in case of issues during the first months of my job. | 68 (32%) | 33 (15%) | 112 (53%) |
| My case worker informed me well about the consequences of finding a job on my welfare benefits. | 42 (20%) | 49 (23%) | 122 (57%) |
| ***Statements regarding the support for job interviews or applications (n=51)*** | | | |
| My contact person was kind enough to wish me luck with my job application(s). | 3 (6%) | 4 (8%) | 44 (86%) |
| My contact person showed interest in how my job interview(s) went. | 2 (4%) | 4 (8%) | 45 (88%) |
| I have learned a lot from the preparations for my job interview(s). | 2 (4%) | 7 (14%) | 42 (82%) |
