# Supplementary material for: Experiences and needs of welfare benefit recipients regarding their welfare-to-work services and case workers
Source: BMC Health Serv Res. 2023 Sep 14;23:990. doi: 10.1186/s12913-023-09954-y (PMC10502984; doi:10.1186/s12913-023-09954-y)
Supplement: Supplementary file 4 — Supplementary Material 4 [file 12913_2023_9954_MOESM4_ESM.docx]

**Appendix 4 – overview of group interviews**

Interview 1

| **Sex** | **Age** | **Education level** | **Period of receiving welfare-to-work services** | **Current job status** |
| --- | --- | --- | --- | --- |
| Female | 52 | High | >12 Months | Part time job + part time education |
| Female | 58 | Low | 2 years | Paid job |
| Female | 38 | Medium | 1,5 years | Education |

Interview 2*

| **Sex** | **Age** | **Education level** | **Period of receiving welfare-to-work services** | **Current job status** |
| --- | --- | --- | --- | --- |
| Female | 30 | High | 4 Months | Unemployed |
| Female | 31 | Medium | 2 Appointments | Unemployed |
| Female | 28 | Medium | 3 Months | Unemployed |
| Female | 31 | Low | 1 Appointment | Unemployed |
| Female | 29 | Medium | 1 Appointment | Unemployed |

* 2 external trainers were present

Interview 3

| **Sex** | **Age** | **Education level** | **Period of receiving welfare-to-work services** | **Current job status** |
| --- | --- | --- | --- | --- |
| Female | 43 | Low | 1 year | Work programme (part of welfare-to-work services) |
| Female | 42 | Low | 5 years | Voluntary work |
| Female | 42 | High | 7 years | Voluntary work |
| Female | 39 | High | 2 years | Voluntary work |

Interview 4

| **Sex** | **Age** | **Education level** | **Period of receiving welfare-to-work services** | **Current job status** |
| --- | --- | --- | --- | --- |
| Male* | 47 | High | >2 years | Unemployed |
| Male | 58 | High | 2,5 months | Self-employed |

* Worked as a case manager in the past
